# Case Report: Local anesthetic systemic toxicity during regional anesthesia in a patient with rheumatoid arthritis

**DOI:** 10.3389/fmed.2026.1758548

**Published:** 2026-02-16

**Authors:** Misa Baic, Miodrag Milenovic, Danilo Obradovic, Relja Lukic, Gordana Dragovic, Bozana Obradovic

**Affiliations:** 1 Department of Anesthesiology, Resuscitation and Intensive Therapy, Institute of Orthopedics “Banjica”, Belgrade, Serbia; 2 University Clinical Center of Serbia, Emergency Center, Center for Anesthesiology and Resuscitation, Belgrade, Serbia; 3 University of Belgrade, Faculty of Medicine, Belgrade, Serbia; 4 Institute of Pathology, University of Belgrade, Faculty of Medicine, Belgrade, Serbia; 5 Clinic for Gynecology and Obstetrics “Narodni Front”, Belgrade, Serbia; 6 Department of Pharmacology, Clinical Pharmacology and Toxicology, University of Belgrade, Faculty of Medicine, Belgrade, Serbia

**Keywords:** central nervous system, levobupivacaine, lidocaine, regional anesthesia, rheumatoid arthritis, toxicity

## Abstract

**Background:**

Local anesthetic systemic toxicity (LAST) is a rare yet potentially life-threatening complication that requires prompt recognition and management. This condition commonly presents with manifestations that involve both the central nervous system (CNS) and the cardiovascular system (CVS). While LAST occurs infrequently during peripheral nerve blocks, its consequences can be severe if left unrecognized.

**Case presentation:**

This article presents a case involving CNS manifestations of LAST in a patient with rheumatoid arthritis (RA). The patient was a 69-year-old female individual who was admitted for evaluation and treatment of a distal radius fracture previously managed at another healthcare facility and subsequently underwent open reduction and internal fixation (ORIF) at our institution. We propose that RA may constitute an additional risk factor for LAST development due to disease-associated alterations in vascular permeability.

**Conclusion:**

Older patients with rheumatoid arthritis may be at increased risk for LAST due to disease-related vascular permeability changes, showing the need for increased vigilance during the administration of regional anesthesia. Early diagnosis and timely administration of intravenous lipid emulsion (ILE) remain critical for the successful management of this potentially life-threatening complication.

## Introduction

Local anesthetic systemic toxicity (LAST) is a serious and uncommon complication occurring after the administration of local anesthetic drugs. LAST is usually presented as central nervous system (CNS) toxicity and/or cardiovascular system (CVS) instability.

CNS toxicity may lead to excitatory symptoms, such as agitation, confusion, and seizures, or inhibitory symptoms, such as mental slowing, reduced level of consciousness, respiratory arrest, and coma. CVS toxicity may present with hyperdynamic features, such as hypertension and tachydysrhythmia, or with depressive manifestations, including hypotension, bradycardia, conduction block, ventricular arrhythmias, and cardiac arrest. In a single-center study conducted in 2014, which included 80,661 patients, Liu et al. reported an overall incidence of LAST of 0.04 per 1,000 peripheral nerve blockades (PNBs) (1). In contrast, Morwald et al. in a 2017 study, which included 238,473 patients who received PNBs, found the overall incidence of LAST to be approximately 1.8 cases per 1,000 PNBs (2). Moreover, a study by Rubin et al. found that the incidence of LAST for shoulder arthroplasty was 4.27 per 1,000 PNBs, which is significantly higher compared to the incidence of LAST following PNBs for hip and knee surgeries (3). It is, however, possible that, due to underreporting and diagnostic errors, the real incidence is higher than currently reported.

Additional risk factors for LAST identified in current data include very young and older patients, reduced muscle mass, chronic comorbidities, pregnancy, and the use of drugs including beta-blockers, digoxin, and calcium channel blockers (4, 5).

First-line treatment for LAST is intravenous lipid emulsion (ILE), which is administered initially as a rapid bolus followed by a continuous infusion based on the patient’s body weight. Continuous monitoring for at least 6 h is recommended to detect and manage these events promptly (6).

Here, we report a case of LAST occurring after an axillary brachial plexus block in a patient with hypertension and seronegative rheumatoid arthritis (RA).

## Case presentation

A 69-year-old female patient with a BMI of 24.7 (1.53 m and 59 kg) was admitted to the hospital for surgical management of a left wrist fracture sustained 2 weeks earlier after a fall on a flat surface. Initially, she was treated at another medical facility, where an orthopedic attempt at reposition was performed and a radius splint was applied.

On admission, a complete preoperative physical examination was performed, including a chest X-ray and ECG, both of which revealed no significant pathological findings. Surgical indication for open reduction and internal fixation (ORIF) of the distal radius was established. Biochemical parameters, complete blood count, thyroid panel, and coagulation screening results were all within normal ranges (Table 1).

**Table 1:** Patient laboratory parameters at admission.

Parameter	Result	Reference range	Unit
White blood cells	10.47	3.70–9.50	10^9^/L
Red blood cells	4.02	3.90–5.20	10^12^/L
Hemoglobin	128	120–153	g/L
Hematocrit	0.383	0.370–0.460	L/L
Platelets	280	140–400	10^9^/L
Glucose	5.17	4.10–5.90	mmol/L
Urea	3.6	2.8–7.2	mmol/L
Creatinine	77	48–96	μmol/L
eGFR	>60		mL/min/1.73m^2^
Total proteins	63	66.8–83.0	g/L
Sodium	144	136–146	mmol/L
Potassium	3.8	3.5–5.3	mmol/L
AST (GOT)	33	0–35	U/L
ALT (GPT)	40	0–34	U/L
aPTT	25.8	24.0–35.0	sec
Prothrombin time	0.99	2.00–3.50	INR
TSH	1.100	0.270–4.200	mIU/L
FT4	21.15	12.00–22.00	pmol/L

The patient’s medical history included osteoporosis and hypertension since 2006 and seronegative rheumatoid arthritis (RA) since 2016. She denied other systemic comorbidities but reported previous orthopedic interventions, osteosynthesis of the pelvis and femur at another hospital. She reported a history of smoking approximately one pack per day and denied any food or drug allergies.

A previous rheumatology report was also provided. Current medications included perindopril/indapamide 10/25 mg and perindopril 10 mg, both taken orally q.d.; fenofibrate 10 mg, q.d.; nadroparin calcium 0.3 mL, administered subcutaneously q.d.; and bromazepam 3 mg, taken orally the night before surgery. Methotrexate was administered at a dose of 7.5 mg once weekly, with the last dose received 25 days before admission.

On the day of surgery, intravenous access was established, oxygen was administered at a flow rate of 2 L/min via face mask, and routine hemodynamic monitoring was performed. On physical examination, the patient’s vital signs were steady. The patient received premedication with midazolam 5 mg administered intramuscularly.

A brachial plexus block was performed using the axillary approach with the aid of a peripheral nerve stimulator (PNS) and a 22-gauge, 10-cm insulated needle. Excellent motor responses were obtained from the median, radial, and ulnar nerves, with a negative aspiration test. Lidocaine 1.3% (20 mL) without adrenaline and levobupivacaine 0.25% (20 mL) were administered. After every 2-4 mL of local anesthetic injection, aspiration was repeated. Verbal communication was maintained throughout the procedure to continuously assess the patient’s mental status and minimize the risk of intraneural injection.

During anesthetic administration, the patient remained hemodynamically stable and communicative, with vital signs within the normal range. Approximately 3 min after the block, the patient reported a sensation of numbness in the arm. Furthermore, 10 min after the block, she developed a fixed, non-dynamic gaze, slowed and incoherent speech, and involuntary movements of the upper limb.

The pupils were of normal size and reacted appropriately to light. Vital signs remained within normal limits: blood pressure 130/80 mmHg, SpO₂ 99% on room air, and heart rate 66/min.

Suspecting LAST, 100 mL of 20% intravenous lipid emulsion (Intralipid®) was administered immediately in the operating room. Although a slightly higher initial bolus (≈1.7 mL/kg) of lipid emulsion was administered, this dose remained within the safe therapeutic range (1.5–2 mL/kg) recommended by the American Society of Regional Anesthesia and Pain Medicine (ASRA) and other guidelines (7–9). The patient was transferred under continuous monitoring to the intensive care unit (ICU) for further observation and was shortly thereafter examined by a neurologist, who noted dysarthria, a fixed gaze, spontaneous breathing, acyanosis, and hemodynamic stability.

After 20 min in the intensive care unit, the neurological symptoms began to resolve, and adequate communication was established with the patient. She was fully oriented but stated that she could not remember the events from the moment the PNB was administered. She reported that at one point, she became aware of her surroundings but was unable to speak. In this patient’s care, considering hemodynamic stability, a continuous infusion of lipid emulsion was not administered.

A complete neurological examination was performed again, and the patient showed no signs of an acute neurological condition, apart from paresthesia in the left arm due to the regional block. During the further course of treatment, the patient was observed in the ICU for the next 24 h, after which she was released hemodynamically stable and with adequate respiratory function. Four days after the LAST episode, the patient successfully underwent surgery under general anesthesia (GA). The perioperative course was uneventful, and she was discharged on the fifth postoperative day for continued home recovery.

## Discussion

LAST is a potentially fatal complication that occurs due to excessive accumulation of local anesthetic in the blood, often caused by accidental intravascular administration, overdose of the local anesthetic, or impaired metabolism or elimination of the drug. LAST can manifest with CNS and/or CVS symptoms, which, if not recognized in time, can quickly reach a point of no return (10). The pathophysiology involves local anesthetics binding to voltage-gated sodium channels, thereby disrupting neuronal and cardiac conduction. The risk and severity of toxicity vary by agent, with bupivacaine being notably more cardiotoxic than lidocaine or ropivacaine due to its higher affinity for and prolonged binding to cardiac sodium channels (11).

The diagnosis and treatment of LAST are guided primarily by expert consensus statements and practice advisories, rather than by robust, high-level clinical practice guidelines. Both clinical protocols and recommendations, including the ASRA algorithm and American Heart Association (AHA) guidelines, although widely adopted in Europe and internationally, are consensus-based and developed from case reports, registry data, and limited clinical trials (12).

This reliance on consensus statements and expert opinion constrains the diagnostic process for LAST in several ways, including the absence of validated diagnostic criteria and the broad spectrum and insidious nature of LAST presentations described in case reviews, which can lead to under-recognition or misdiagnosis, especially in perioperative settings where symptoms may overlap with other conditions.

LAST is primarily a clinical diagnosis, based on the temporal relationship between local anesthetic administration and the development of characteristic CNS and/or CVS symptoms, in accordance with established consensus guidelines, such as those of the ASRA and AHA (10, 11).

Symptoms of LAST typically follow a biphasic course: CNS symptoms often precede cardiovascular toxicity and include perioral numbness, facial tingling, metallic taste, auditory changes, agitation, confusion, dizziness, dysarthria, and seizures. CNS excitation may progress to CNS depression, including obtundation and coma (12).

Cardiovascular symptoms range from mild hypertension and tachycardia to severe manifestations such as conduction delays, bradycardia, hypotension, ventricular arrhythmias, asystole, and cardiac arrest. Cardiovascular collapse may occur abruptly, especially with highly lipophilic agents such as bupivacaine (11).

Our patient developed acute neurological symptoms—numbness, auditory changes, agitation, confusion, and dysarthria—within minutes of receiving a mixture of local anesthetics (levobupivacaine and lidocaine) via an axillary approach to the brachial PNB. These manifestations are consistent with the early central nervous system signs of LAST. The absence of hemodynamic instability does not exclude LAST, as CNS symptoms may occur in isolation, especially in the initial phase (13).

In half of the cases analyzed by Gregorio di Guido et al. in 2009, toxicity symptoms manifested within the initial 50 s, and three-quarters of cases displayed clinical signs of toxicity within the first five min. Another finding of this study was that half of the patients with LAST were women (14). Late-onset effects following local anesthesia administration are thought to be more significantly influenced by factors that affect how the anesthetic is absorbed.

The absorption rate can be influenced by multiple factors, including the type of medication selected, dosage amount, the rate of administration, the presence of vasoactive adjuvants, the patient’s age and comorbidities, local blood vessel permeability and blood flow in the area surrounding the nerve, how well the medication binds to local tissue, and the drug’s liposolubility properties (15).

The first CNS symptoms, sensations of numbness in the arm, in our patients appeared 3 min after anesthetic administration, while slow and incoherent speech; a fixed, non-dynamic gaze; involuntary movements of the upper limb, and memory loss occurred approximately 10 min after local anesthetic administration. The total doses administered, 260 mg of lidocaine and 50 mg of levobupivacaine, were below the recommended toxic dose limits (16). Although ultrasound guidance is currently preferred, peripheral nerve stimulator techniques remain acceptable when performed with repeated aspiration and continuous verbal monitoring to minimize intravascular injection. Nevertheless, several pharmacologic and patient-related factors may explain the occurrence of toxicity despite adherence to guideline-recommended dosing. These factors include the combined use of two amide local anesthetics with potentially additive systemic toxicity, increased vascular absorption associated with the axillary block site, age-related pharmacokinetic changes, and possibly reduced plasma protein binding, as suggested by slightly decreased albumin levels (17).

Current literature predominantly focuses on well-established risk determinants, including patient demographics (age, sex), pharmacologic parameters (dose, concentration, specific agent), technical factors (route and site of administration), and organ system dysfunction (hepatic or renal impairment). Additional risk factors for this patient included older age, female sex, and the additional toxicity from combining two local anesthetic agents. Although this patient has RA as a chronic comorbidity, there are no documented cases in the medical literature specifically describing LAST in patients with RA. Large retrospective studies and case series on LAST, including those analyzing risk factors and patient comorbidities, do not identify RA as a reported comorbidity, nor do they analyze its presence as a risk modifier The enhanced vascular permeability, endothelial dysfunction, and altered protein binding associated with chronic inflammation warrant consideration when assessing individual patient risk, and direct evidence linking RA to an increased risk of LAST remains insufficient (3, 18).

In this case, the immediate administration of 100 mL of 20% intravenous lipid emulsion (ILE) for suspected LAST was consistent with current evidence-based recommendations. Early use of ILE is supported by consensus guidelines and expert opinion as a first-line intervention for neurological manifestations of LAST, particularly when central nervous system toxicity occurs shortly after a peripheral nerve block with amide local anesthetics such as lidocaine and levobupivacaine Prompt recognition and treatment of LAST are prioritized to prevent progression to seizures or cardiovascular collapse (7, 10). The decision not to pursue further immediate neurological evaluations (such as urgent neuroimaging, EEG, or additional neurological consultation) was based on several factors: The strong temporal relationship between local anesthetic administration and early symptom onset, the classic clinical presentation of LAST, and the absence of persistent focal neurological deficits or hemodynamic instability. Neurological symptoms, including a fixed gaze, incoherent speech, and involuntary movements, developed within min of the block and resolved rapidly following lipid emulsion therapy. In accordance with current recommendations, immediate neuroimaging is not routinely indicated in the absence of persistent or progressive neurological findings.

## Conclusion

Local anesthetic systemic toxicity is a rare but potentially life-threatening complication that requires prompt recognition and management. This case illustrates that LAST may occur despite adherence to recommended dosing guidelines, particularly in the presence of multiple pharmacologic and patient-related risk factors. The patient developed clinical manifestations of LAST, including a fixed, non-dynamic gaze, slowed and incoherent speech, and involuntary upper limb movements, secondary to levobupivacaine and lidocaine toxicity. Treatment with 20% intravenous lipid emulsion resulted in complete symptom resolution without further complications. Consideration of patient risk factors, such as age, sex, and presence of comorbidities, is important to prevent or mitigate adverse outcomes associated with LAST.

## Data Availability

The original contributions presented in the study are included in the article/Supplementary material, further inquiries can be directed to the corresponding author.
